# Association Between Nerve Block Injection and Short-Term Outcomes in Patients With Acute Low Back Pain: Findings From the Acute Low Back Pain Study, a Prospective Observational Study

**DOI:** 10.7759/cureus.88955

**Published:** 2025-07-29

**Authors:** Ryota Kawai, Eriko Komiya, Hisashi Date, Takashi Kamezawa, Yuta Nonomiya, Hisako Yoshida, Ayumi Shintani

**Affiliations:** 1 Department of Medical Statistics, Osaka Metropolitan University Graduate School of Medicine, Osaka, JPN; 2 Department of Orthopedic Surgery, Osaka Metropolitan University Graduate School of Medicine, Osaka, JPN; 3 Department of Anesthesiology, Sendai Pain Clinic Center, Sendai, JPN; 4 Department of Anesthesiology, Japan Society of Pain-clinic Practitioners, Tokyo, JPN; 5 Department of Anesthesiology, Kamezawa Clinic, Aichi, JPN; 6 Department of Smart Data and Knowledge Services, Deutsches Forschungszentrum für Künstliche Intelligenz, Kaiserslautern, DEU

**Keywords:** acute low back pain, nerve block injection, pain disability assessment scale, real-world, visual analog scale

## Abstract

Background

Acute low back pain is a leading cause of disability worldwide. While clinical guidelines offer general management strategies, real-world evidence on nerve block therapy for acute low back pain in primary care remains limited.

Methods

We conducted a prospective, multicenter observational cohort study at 19 private pain clinics in Japan from June 2021 to March 2022. Patients with acute low back pain onset within the past seven days were enrolled and followed for seven days. Clinical decisions, including nerve block administration, were made at the physician’s discretion. Patient-reported outcomes were collected via web- or paper-based questionnaires and compared between patients who received nerve block injections and those who did not. The primary outcome was the change in Pain Disability Assessment Scale (PDAS) scores from baseline to Day 3; secondary outcomes included PDAS changes on Day 7 and Visual Analog Scale (VAS) scores for seven consecutive days. Multivariable regression models were used to adjust for confounding factors.

Results

A total of 567 patients were analyzed (523 injected; 44 non-injected). Among patients with baseline PDAS scores ≥ 45, those who received injections showed significantly greater improvement on Day 3. The mean differences (95％ confidence interval (CI) ) in PDAS scores were -6.42 (-12.48 to -0.36) (*P* = 0.038) at a baseline score of 45, -11.95 (-21.35 to -2.55) (*P* = 0.013) at 50, and -17.54 (-31.01 to -4.07) (*P* = 0.011) at 55. VAS scores were significantly lower in patients with the injection on Day 1 (mean difference (95% CI): -0.70 (-1.37 to -0.04)) and Day 3 (-0.76 (-1.44 to -0.08)), with no significant differences beyond Day 3.

Conclusion

Nerve block therapy was associated with faster pain relief and earlier functional improvement in acute low back pain patients, particularly among those with greater baseline disability. These findings provide meaningful real-world evidence. Given the observational design and the fact that treatment decisions were made at the discretion of attending physicians, there remains a potential for selection bias. Further well-designed studies are needed to validate these findings and guide optimal treatment strategies for acute low back pain.

## Introduction

Low back pain (LBP) is one of the most prevalent health problems worldwide and a leading cause of disability, affecting approximately 7.5% of the global population at any given time [1, 2]. A substantial proportion of patients are diagnosed with non-specific low back pain (NSLBP), in which no definitive structural pathology can be identified [3, 4]. While extensive research has focused on the etiology and treatment of NSLBP, particularly in chronic stages, evidence regarding the management of acute low back pain remains limited [5-7]. Current clinical practice guidelines commonly recommend pharmacological therapy, exercise therapy, and cognitive behavioral therapy for the management of acute low back pain [8-10]. However, these recommendations are often based on data from controlled environments, such as large hospitals or academic centers, which may not fully reflect the realities of everyday clinical practice. In fact, many patients with acute low back pain initially seek care at small hospitals or private clinics, where healthcare professionals provide frontline management of this common condition. Despite their central role in the treatment of acute low back pain, these settings are underrepresented in clinical research, and there is a critical need to generate evidence from real-world practices.

Among various treatment options, nerve block therapy is frequently used in small clinical settings as a pragmatic intervention for pain relief in acute low back pain. Although several studies have evaluated its effectiveness in patients with chronic low back pain [11-14], robust evidence for its use in acute settings is still lacking.

Commonly used nerve block types include facet joint injections, medial branch blocks, epidural steroid injections (via interlaminar, transforaminal, and caudal approaches), and selective nerve root blocks. The most frequently administered agents are local anesthetics (e.g., lidocaine, bupivacaine), either alone or in combination with corticosteroids (e.g., betamethasone, triamcinolone). Nevertheless, detailed information on injection dosage is often inconsistently reported across studies, and standardized protocols remain limited.

Among these previous studies, Riew et al. conducted a randomized, double-blind trial on selective nerve blocks for lumbar spine disorders [14]. Their five-year follow-up showed that 29 out of 55 patients avoided surgery, and 17 demonstrated significant clinical improvement. However, because the study included only patients with chronic low back pain, the applicability of these findings among patients with acute low back pain remains uncertain.

Most RCTs in LBP are focused on chronic or non-specific LBP. Those targeting acute low back pain and those involving nerve blocks remain limited [3-4,11-13]*. *The OPAL trial examined the effectiveness of pharmacological therapy for acute spinal pain. They found no meaningful clinical benefit compared to placebo while reporting non-ignorable adverse events [15]. This study highlighted the limited role of pharmacological therapy in the management of acute non-specific spinal pain and raised questions about its routine use.

In contrast, our observational study examined the real-world use of block injection among patients with acute low back pain, especially in the setting of primary care in Japan. By focusing on real-world treatment decisions and outcomes such as pain relief and functional improvement, our study provides complementary evidence that reflects the complexity of patient care in routine settings and may help inform future trials or clinical recommendations.

To address this knowledge gap, we conducted a prospective observational study in collaboration with private practitioners affiliated with the Japan Society of Pain-clinic Practitioners. The primary aim of this study is to evaluate the association between nerve block therapy and short-term changes in pain and functional outcomes in patients with acute low back pain. By leveraging data from actual clinical settings, this study provides real-world insights that may inform future research and contribute to the development of clinical guidelines for the management of acute low back pain.

## Materials and methods

Study design and setting

This prospective, multicenter observational cohort study was conducted in 19 facilities affiliated with the Japan Society of Pain-clinic Practitioners** **between June 2021 and March 2022. Ethical approval was obtained from an independent ethical committee (Osaka Metropolitan University) in June 2021 (Approval number: 2021-103), and it was accepted by all the sites: Sendai Pain Clinic Center, Kamezawa Clinic, Umesato Itamitonaikano Clinic, Shiga Rhematology and Orthopedic Clinic, Yamamoto Clinic, Nakamura Pain Clinic, Terada Pain Clinic, Suetsugu iin, Makiminato Pain Clinic, Miyazawa Pain Clinic, Hase Pain Clinic, Tamura Pain Clinic, Kakihara Pain Clinic, Shiraishi Orthopedic Clinic, Kanayama Pain Clinic, Yamanote Pain Clinic, Kagurazaka-iin, Kameido Satou Noriko Clinic and Hakata Pain Clinic.

In this study, patients with acute low back pain who met the inclusion and exclusion criteria at the collaborating institutions were provided with a written explanation of the study. Written informed consent was obtained from all participants. Participants completed a daily questionnaire regarding their back pain status for seven consecutive days starting on the day of treatment. Data was collected using two methods: web-based or paper-based questionnaires. For the web-based questionnaire, we used the LINE mobile messaging application - a widely used communication platform in Japan - to avoid collecting personally identifiable information such as email addresses. Patients who consented to using LINE were contacted via a secure one-on-one chat with a LINE Bot, which automatically delivered daily links to access the Research Electronic Data Capture (REDCap; Vanderbilt University, Nashville, USA) questionnaire from baseline to Day 7. Participants entered their data, including Visual Analog Scale (VAS) score [16] and Pain Disability Assessment Scale (PDAS) scores [17], directly into the system. If a patient did not have a mobile device or declined to use the LINE application, they were asked to complete the equivalent questionnaire on paper. For paper-based responses, data from the day of treatment were entered by the personnel at the collaborating institution, and patients’ responses for their back pain status for subsequent days were mailed to the data-coordinating center for data entry into REDCap.

We obtained official permission from the LINE Corporation to use the LINE platform for our study. This official LINE account enabled communication with participants without collecting personal information, such as LINE IDs or phone numbers. The study followed the Strengthening the Reporting of Observational Studies in Epidemiology (STROBE) reporting guideline.

Participants

We enrolled patients who had all of the following: (1) age ≥ 20 years at the time of informed consent; (2) onset of acute low back pain within the past 7 days; (3) pain of sufficient severity to interfere with daily activities; and (4) provision of written informed consent for participation in this study. Patients were then excluded if they had at least one of the followings: (1) uncontrolled diabetes mellitus (glycated hemoglobin (HbA1c) ≥ 8.0%); (2) severe renal impairment (estimated glomerular filtration rate (GFR) ≤ 30 ml/min); (3) lack of independence in daily living before their symptom onset; (4) other injuries in addition to LBP; (5) suspected of vertebral fractures; (6) history of cancer; (7) trauma due to traffic accidents; and (8) treatment under the industrial accident compensation insurance.

Exposure

As this was a non-interventional observational study, the decision to perform a nerve block - including the type of block, anatomical site, injectates used, and use of guidance devices - was made by the treating physicians based on each patient's clinical condition. Corresponding data on the type of nerve block (e.g., epidural block, facet joint block, trigger point injection), injectates (e.g., local anesthetics, corticosteroids, normal saline), and guidance methods (e.g., ultrasound, fluoroscopy) were obtained from routine clinical records.

Variables

Baseline variables included demographic and clinical characteristics (age, sex, height, weight, living arrangement (e.g., living alone or living with others), and history of acute low back pain), occupational factors (employment status, workplace at home, means of commuting, commuting time >30 minutes, and type of work), medication use (e.g., prescribed medications, nonsteroidal anti-inflammatory drugs (NSAIDs), non-opioid analgesics, acetaminophen, antidepressants, Kampo medicine, and topical agents), comorbidities (e.g., musculoskeletal disorders, malignancies, and other chronic conditions), and baseline pain and disability(e.g., PDAS and VAS scores).

Outcome

The primary outcome was the change in PDAS score from baseline to Day 3 after the treatment, compared between the groups. The secondary outcomes included the following: (1) the change in PDAS score from baseline to Day 7; and (2) the daily change in VAS scores from baseline to each day up to Day 7.

Statistical analysis

Baseline characteristics of the patients were summarized as medians with interquartile range (IQRs) for continuous variables, and frequencies with percentages for categorical variables.

To examine the effect of nerve block on the change in PDAS scores from baseline to Day 3 after the treatment, we conducted multivariable linear regression analysis, including the following explanatory variables: age, sex, the baseline PDAS score, history of acute low back pain, injection status, and cross-product terms between the baseline PDAS score and injection status. A restricted cubic spline function with three knots was applied to the baseline PDAS score to assess the non-linear effect. A similar model was employed to analyze the change in PDAS scores from baseline to Day 7 following treatment.

To examine the effect of nerve block injection on VAS scores from Day 1 to Day 7, we conducted a multivariable mixed-effects regression model including the following explanatory variables: age, sex, the baseline VAS score, history of acute low back pain, injection status, and cross-product terms between baseline VAS and injection status.

Missing values for outcomes and covariates were addressed using multiple imputation methods using predictive mean matching [18]. To examine the effect of nerve block injection on VAS scores from Day 1 to Day 7, missing data were handled using a mixed model for repeated measures (MMRM), which has been shown to provide valid inferences under the missing at random (MAR) assumption without the need for explicit imputation. Mallinckrodt et al. demonstrated that MMRM controls Type I error more accurately and yields less biased estimates compared to last observation carried forward (LOCF), even in the presence of incomplete longitudinal data. Therefore, we did not perform additional imputation procedures [19].

All statistical analyses were conducted using R version 4.4.1 (R Foundation for Statistical Computing; R Foundation, Vienna, Austria) software [20]. Statistical significance was defined as a p-value of < 0.05.

## Results

**Figure 1:**
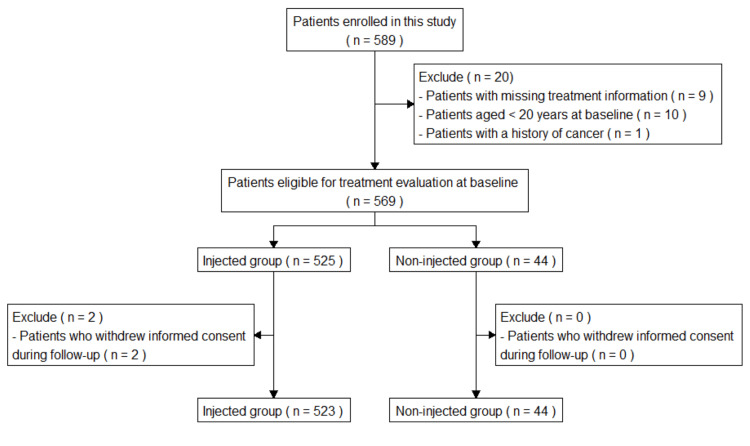
Flow diagram of patient enrollment and inclusion in the analysis. A total of 589 patients were enrolled in the study across 19 participating facilities. Twenty patients were excluded due to missing treatment information, age <20 years at baseline, or a history of cancer. Among the 569 eligible patients, 525 received a nerve block injection and 44 did not. Two patients in the injected group withdrew informed consent during follow-up and were excluded, resulting in a final analytic sample of 567 patients.

A total of 589 patients were enrolled from 19 participating facilities in this observational study (Figure 1). Of these, 20 patients were excluded due to missing treatment information (n = 9), being under 20 years of age at baseline (n = 10), or having a history of cancer (n = 1). The remaining 569 patients met the eligibility criteria and were not excluded. Among them, 525 patients received a nerve block injection at baseline, while 44 patients did not. During follow-up, two patients in the injected group withdrew informed consent and were excluded, resulting in a final analytic sample of 567 patients (523 in the injected group and 44 in the non-injected group).

This notable imbalance in sample size between groups (523 vs. 44) should be taken into consideration when interpreting between-group comparisons, as it may affect statistical power and precision of the estimates. The baseline characteristics of the study population are summarized in Table 1. No formal statistical testing was performed between groups, in line with the observational nature of the study. Overall, the two groups were generally similar in terms of demographic and clinical characteristics, although slight differences were noted in prior history of acute low back pain and in the types of medications prescribed at baseline.

**Table 1:** Baseline characteristics of patients with acute low back pain. Demographic, clinical, and treatment-related characteristics of patients in the injected (n = 523) and non-injected (n = 44) groups. Values are presented as median (interquartile range) for continuous variables and number (percentage) for categorical variables. Abbreviations: NSAIDs, non-steroidal anti-inflammatory drugs; PDAS, Pain Disability Assessment Scale; VAS, Visual Analog Scale; IQR, inter-quantile range; ALBP, acute low back pain

Variables		Non-injected group (n = 44)	Injected group (n = 523)	Missing (%)
Age, years		47.0 (40.5, 56.3)	49.0 (40.0, 56.0)	0.0
Male		29 (65.9)	341 (65.2)	0.0
Height, cm		165.5 (158.8, 171.3)	168.0 (160.0, 174.0)	0.2
Weight, kg		64.5 (56.0, 77.3)	67.5 (58.0, 75.0)	0.2
Living alone		7 (15.9)	86 (16.4)	0.0
Living with someone who provides support		35 (94.6)	414 (95.0)	16.6
Living with someone who needs care		12 (32.4)	140 (32.1)	16.6
History of ALBP		27 (61.4)	430 (82.2)	0.0
Employed		36 (81.8)	460 (88.3)	0.4
Workplace at home		1 (2.8)	36 (7.8)	12.5
Means of commuting	Walking or bicycle	1 (2.9)	45 (10.6)	19.0
	Car or taxi	31 (88.6)	351 (82.8)	
	Public transportation	3 (8.6)	28 (6.6)	
Commuting time > 30 minutes		10 (28.6)	104 (24.5)	19.0
Type of work	Desk work or seated tasks	10 (27.8)	121 (26.4)	12.9
	Standing work	13 (36.1)	205 (44.8)	
	Both desk and standing work	13 (36.1)	132 (28.8)	
Prescription provided		43 (97.7)	411 (78.6)	0.0
	NSAIDs	40 (90.9)	320 (61.2)	
	Non-opioid analgesics	0 (0.0)	17 (3.3)	
	Acetaminophen	1 (2.3)	13 (2.5)	
	Antidepressants	0 (0.0)	1 (0.2)	
	Kampo medicine	1 (2.3)	97 (18.5)	
	Topical medications	33 (75.0)	190 (36.3)	
Presence of treated comorbidities		10 (22.7)	128 (24.5)	0.2
	Musculoskeletal disorders	1 (2.3)	34 (6.5)	
	Malignancy	0 (0.0)	1 (0.2)	
	Others	9 (10.5)	98 (18.7)	
Anti-inflammatory analgesics		3 (6.8)	36 (6.9)	0.0
Oral steroid medications		0 (0.0)	2 (0.4)	0.0
The baseline PDAS score		22.0 (14.8, 38.5)	34.0 (24.8, 43.0)	0.5
The baseline VAS		6.6 (4.7, 7.6)	7.5 (6.5, 8.4)	0.7

Information regarding the type of nerve block, injectates, and guidance methods is summarized in Table 2. Epidural blocks were the most frequently performed procedure, accounting for approximately two-thirds of the patients. Most injections involved local anesthetics and corticosteroids. Guidance devices were used in nearly half of the procedures, with ultrasound and fluoroscopy being the primary methods.

**Table 2:** Summary of nerve block procedures and injectates This table presents detailed information on the types of nerve blocks performed, the injectates used, and the guidance devices used. Percentages are calculated based on the total number of patients who received a nerve block (n = 523). Some patients received more than one type of injectate or guidance method. Importantly, "Normal saline only" refers to procedures in which normal saline was used without any concomitant use of local anesthetics or corticosteroids. Procedures involving saline for dilution or as part of the loss-of-resistance technique were not included in this category. Abbreviations; CT, computed tomography;

Variables		% (n)	Missing (%)
Type of nerve block			0.0
	Facet joint block	26.8% (140)	
	Epidural block	66.2% (346)	
	Trigger point injection	15.9% (83)	
	Others	11.1% (58)	
Injectates			0.0
	Local anesthetic	99.4% (520)	
	Corticosteroid	84.9% (444)	
	Normal saline only	0.2% (1)	
	Others	3.1% (16)	
Use of guidance device		45.5% (238)	0.0
Type of guidance device	Ultrasound guidance	24.7% (129)	0.0
	Fluoroscopic guidance	23.1% (121)	
	Peripheral nerve stimulator	0.0% (0)	
	CT guidance	0.0% (0)	
	Others	0.0% (0)	

**Figure 2:**
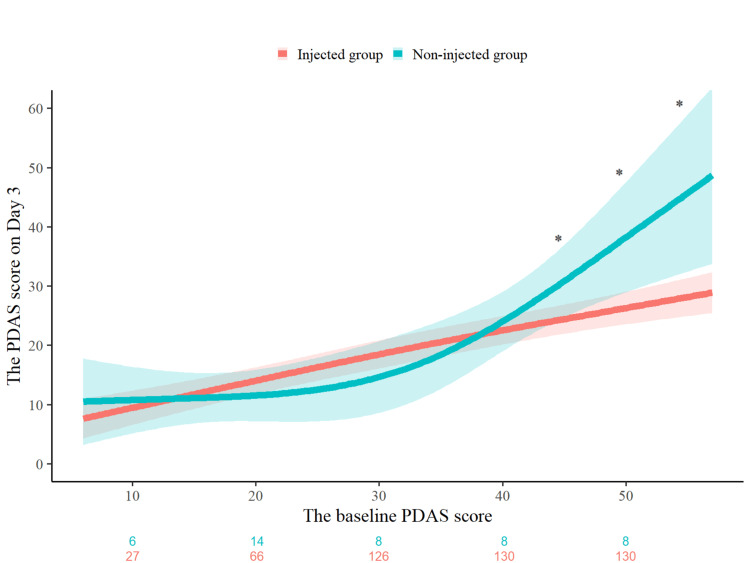
Association between the baseline PDAS score and the PDAS score on Day 3 by injection status. Adjusted PDAS scores on Day 3 are shown by baseline PDAS scores, stratified by injection status. The estimates were derived from a multivariable linear regression model that included the same explanatory variables: age, sex, baseline PDAS score, history of acute low back pain, injection status, and the cross-product term between baseline PDAS and injection status. Shaded areas represent 95% confidence intervals. A significant interaction was observed (*P* for interaction = 0.043), with a notable increase in PDAS score in the non-injected group at baseline PDAS ≥ 30. * indicates a significant difference between the injected and non-injected groups (*P* < 0.05). Participant counts for each group by baseline PDAS score are indicated below the x-axis. Abbreviations: PDAS, Pain Disability Assessment Scale

Figure 2 shows the adjusted association between the baseline PDAS scores and PDAS scores on Day 3, stratified by injection status. The model included the following explanatory variables: age, sex, the baseline PDAS score, history of acute low back pain, injection status, and cross-product terms between the baseline PDAS score and injection status. A sharp increase in the PDAS score on Day 3 was observed among patients in the non-injected group when the baseline PDAS score exceeded approximately 30. A statistically significant interaction was noted between the baseline PDAS score and injection status (*P* for interaction = 0.043), with significant group difference emerging at baseline PDAS scores of 45 points or higher. This finding was further supported by the stratified analysis shown in Table 3, which demonstrates that the mean difference in the PDAS scores on Day 3 between injected and non-injected groups became statistically significant at baseline PDAS scores of 45 points or higher. For instance, at a baseline PDAS score of 55, the adjusted mean difference reached -17.54 points (95% CI: -31.01 to -4.07; *P* = 0.011).

**Table 3:** The PDAS score on Day 3 stratified by baseline injection status. Adjusted mean differences in the PDAS scores on Day 3 between injected and non-injected groups, stratified by baseline PDAS scores (40 to 55). The estimates are derived from a multivariable linear regression model that included the following explanatory variables: age, sex, baseline PDAS score, history of acute low back pain, injection status, and the cross-product terms between baseline PDAS and injection status. Abbreviations: PDAS, Pain Disability Assessment Scale; CI, confidence interval.

	Mean difference (95% CI)	*P-*value
The baseline PDAS score = 40	-1.48 (-6.35 to 3.39)	0.551
The baseline PDAS score = 45	-6.42 (-12.48 to -0.36)	0.038
The baseline PDAS score = 50	-11.95 (-21.35 to -2.55)	0.013
The baseline PDAS score = 55	-17.54 (-31.01 to -4.07)	0.011

**Figure 3:**
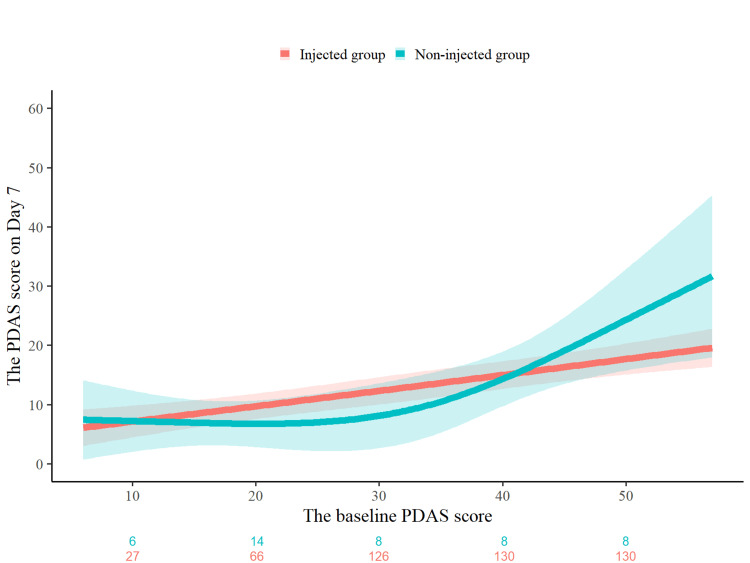
Association between the baseline PDAS score and the PDAS score on Day 7 by injection status. Adjusted PDAS scores on Day 7 are plotted against THE baseline PDAS score, stratified by injection status. The estimates were derived from a multivariable linear regression model that included the same explanatory variables as in Figure 2: age, sex, baseline PDAS score, history of acute low back pain, injection status, and the cross-product term between baseline PDAS and injection status. Although the interaction was not statistically significant (*P* for interaction = 0.174), a similar trend of higher PDAS scores in the non-injected group at higher baseline PDAS values was observed. Participant counts for each group by baseline PDAS score are indicated below the x-axis. Abbreviations: PDAS, Pain Disability Assessment Scale

Similarly, Figure 3 illustrates the association between the baseline PDAS scores and the PDAS scores on Day 7. While a similar trend was observed - namely, greater increases in the non-injected group at higher baseline PDAS levels - the interaction did not reach a statistically significant level (*P* for interaction = 0.174). Although the interaction term was not statistically significant overall, Table 4 presents the differences in the PDAS scores on Day 7, stratified by baseline PDAS level. The estimated mean difference reached -10.52 points at a baseline PDAS score of 55 (95% CI: -22.84 to 1.80; *P* = 0.094), suggesting a potential trend favoring the injected group at higher baseline disability levels.

**Table 4:** The PDAS score on Day 7 stratified by baseline injection status. Adjusted mean differences in the PDAS scores on Day 7 between injected and non-injected groups, stratified by baseline PDAS scores (40 to 55). The estimates were derived from a multivariable linear regression model that included the same explanatory variables as in Table 3: age, sex, baseline PDAS score, history of acute low back pain, injection status, and the cross-product term between baseline PDAS and injection status. Although no comparisons reached statistical significance, a trend toward lower scores in the injected group was observed at higher baseline PDAS levels. Abbreviations: PDAS, Pain Disability Assessment Scale; CI, confidence interval.

	Mean difference (95% CI)	*P-*value
The baseline PDAS score = 40	0.66 (-3.85 to 5.16)	0.775
The baseline PDAS score = 45	-2.77 (-8.36 to 2.82)	0.331
The baseline PDAS score = 50	-6.62 (-15.24 to 1.99)	0.132
The baseline PDAS score = 55	-10.52 (-22.84 to 1.80)	0.094

**Figure 4:**
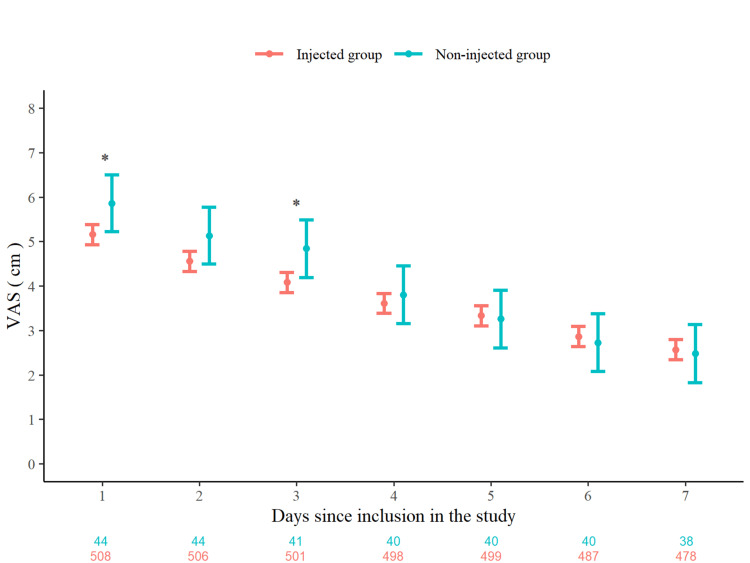
Change in VAS score over 7 days by injection status. Daily mean VAS scores (in cm) are plotted from Day 1 to Day 7, comparing injected and non-injected groups. The estimates were derived from a multivariable mixed-effects regression model that included the following explanatory variables: age, sex, baseline VAS score, history of acute low back pain, injection status, and the cross-product terms between baseline VAS and injection status. Error bars represent 95% confidence intervals. VAS scores were significantly lower in the injected group on Days 1 and 3. * indicates a significant difference between the injected and non-injected groups (*P* < 0.05). Participant counts for each group at each timepoint are indicated below the x-axis. Abbreviation: VAS, Visual Analog Scale

Figure 4 shows the change in VAS over the 7-day period, comparing the injected and non-injected groups. VAS scores were significantly lower in the injected group on Days 1 and 3. These results are consistent with Table 5, which shows that the between-group differences in VAS scores were statistically significant only on Days 1 and 3, with no clear differences observed on subsequent days.

**Table 5:** Between-group differences in VAS score over 7 days. Adjusted mean differences in daily VAS scores (cm) between injected and non-injected groups from Day 1 to Day 7. The estimates were derived from a multivariable mixed-effects regression model that included the following explanatory variables: age, sex, baseline VAS score, history of acute low back pain, injection status, and the cross-product terms between baseline VAS and injection status. Statistically significant differences were observed on Days 1 and 3 only. Abbreviations: VAS, Visual Analog Scale; CI, confidence interval.

	Mean difference (95% CI)
Day 1	-0.70 (-1.37 to -0.04)
Day 2	-0.57 (-1.24 to 0.09)
Day 3	-0.76 (-1.43 to -0.08)
Day 4	-0.19 (-0.87 to 0.48)
Day 5	0.07 (-0.61 to 0.75)
Day 6	0.14 (-0.54 to 0.82)
Day 7	0.09 (-0.60 to 0.77)

## Discussion

In this prospective, multicenter observational study conducted across 19 primary care pain clinics, we investigated the real-world association between nerve block therapy and clinical outcomes in patients with acute low back pain. We compared outcomes between patients who received a nerve block at baseline and those who did not, focusing on daily pain and disability scores during the following seven days. Although return to work was not directly assessed in this study, our findings of more rapid pain relief in the nerve block group may have implications for functional recovery, including earlier resumption of work activities. A previous randomized controlled trial reported that patients who maintained normal daily activities returned to work in an average of 4.1 days, whereas those prescribed bed rest required 7.5 days to return [21]. These findings suggest that most patients recover within a week, even without intervention. Nevertheless, for patients who require time off due to pain, symptom relief during the initial few days becomes clinically meaningful. In our study, the VAS scores on Day 1 were significantly lower in the injected group, suggesting that patients who received a nerve block may have been able to resume work earlier, regardless of work setting. Given that acute low back pain often causes marked functional limitations during the first few days, early pain relief, particularly among patients with higher baseline VAS scores, may critically influence the ability to return to work, even in non-physical job settings.

Previous studies investigating nerve block therapy have largely focused on chronic low back pain. For example, Manchikanti et al. [11] and Staal et al. [13] demonstrated favorable outcomes in patients with chronic low back pain receiving injection-based interventions. However, the generalizability of these findings to the acute setting remains unclear. A Cochrane review by Staal et al. reported limited evidence supporting the use of injection therapy for subacute and chronic low back pain, and no strong evidence for or against its use in acute cases [22]. Our study helps to fill this gap by providing data specific to acute low back pain, with a focus on the short-term association between nerve block injections and symptom improvement in the early phase of symptom onset.

More recently, Cohen et al. [12] emphasized the importance of patient selection and timing of intervention when considering epidural injections. Similarly, our findings suggest that nerve block therapy may be particularly beneficial for patients with greater baseline disability, as evidenced by the significant interaction observed between baseline PDAS scores and injection status. This aligns with the concept that patients with more severe functional limitations may derive greater benefit from early intervention, as demonstrated in a previous study of acute low back pain patients at high risk for chronicity [23].

Another strength of our study is its real-world implementation in primary care clinics. Although acute low back pain is frequently encountered in daily primary medical practice, research from academic institutions often focuses on chronic or complex pain conditions, leaving acute low back pain underrepresented. By collaborating with private pain clinics, this observational study addresses that gap and provides practical, real-world insights into the association between nerve block therapy and clinical outcomes in early-stage acute low back pain.

Our study has several methodological strengths. First, we successfully collected patient-reported outcomes for seven consecutive days - a notable achievement, as many clinical studies face difficulties in follow-up once symptoms begin to improve. By combining mobile application-based and paper-based data collection methods, we minimized barriers to participation and enabled continued data capture outside the clinic. This approach provided valuable evidence on the trajectory of pain relief following treatment. Second, despite the challenges imposed by the COVID-19 pandemic, which restricted patient enrollment in many studies, our multicenter design involving 19 clinics allowed us to recruit a sufficiently large and diverse sample. This enhanced both the generalizability of our findings and demonstrated the feasibility of collaborative research in private practice settings.

Our results provide important evidence regarding the potential benefits of nerve block therapy in managing acute low back pain. Notably, PDAS scores improved more among patients who received nerve block injections, particularly among those with higher baseline disability. This suggests that nerve block therapy may be more beneficial for patients experiencing more severe functional limitations. Furthermore, the VAS scores on Days 1 and 3 highlight the potential for short-term pain relief through early intervention. However, the lack of significant between-group differences after Day 3 suggests that the analgesic effects of a single nerve block injection therapy might be needed to maintain clinical benefit.

The observed heterogeneity in treatment response underscores the need for further investigation into factors that may influence outcomes, such as patient demographics, comorbidities, and variations in medication or injection techniques. Identifying such factors could contribute to more tailored and effective therapeutic strategies.

From a broader perspective, our findings contribute to the growing body of evidence supporting the role of nerve block therapy in pain management. While much of the existing literature has focused on chronic low back pain, our results suggest that nerve block therapy may also be effective in acute presentations. This is consistent with the broader emphasis on early and effective pain control to prevent symptom persistence and long-term disability factors that are associated with increased healthcare utilization and reduced quality of life.

Limitations

Several limitations of this study should be acknowledged. First, the non-randomized allocation of treatment limits the internal validity of between-group comparisons. The decision to administer nerve block injections was left to the discretion of the attending physician, which may have introduced indication bias. For instance, patients with more severe pain or functional impairment may have been more likely to receive injections. This is supported by baseline differences in VAS scores and NSAID use between groups, suggesting possible disparities in symptom severity and prior treatment exposure. In addition, the substantial sample size imbalance between groups (523 vs. 44) may have limited the statistical power and precision of between-group comparisons, particularly for the non-injected group. Moreover, the small number of patients in the non-injected group further limits the statistical power and robustness of comparisons. Nevertheless, these imbalances may also reflect the realities of clinical practice, where treatment decisions are often individualized rather than guideline-driven. Furthermore, because detailed procedural information on nerve block therapy - such as the specific technique, anatomical target, and dosage - was not consistently documented across all sites, we were unable to fully account for variability in injection practices. In addition, potential differences in physicians' clinical experience and decision-making criteria, which were not measured in this study, may have further contributed to variability in treatment delivery and outcomes. These limitations may affect the reproducibility and interpretability of the findings. In this context, nevertheless, the study still provides valuable insight into real-world treatment patterns and outcomes in everyday clinical settings.

Second, although all participants had acute low back pain of less than 7-day duration at enrollment, we did not adjust for the exact number of days since symptom onset. Even within the acute phase, variations in timing may influence pain trajectories and treatment response. Future studies should account for this temporal factor to improve the precision of outcome assessment.

Third, reliance on patient-reported outcomes introduces the possibility of reporting bias. For example, patients receiving block injections may have had higher expectations of treatment benefit, which could have influenced subjective ratings of pain and disability. Future studies could incorporate objective measures, such as imaging or physical performance measures, to complement patient-reported data.

Finally, the follow-up period was limited to seven days, preventing assessment of longer-term outcomes such as recurrence, sustained functional improvement, or healthcare utilization.

## Conclusions

In conclusion, although this study has limitations, it provides valuable real-world evidence supporting the use of nerve block injections for acute low back pain. Given the observational nature of the study, the findings should be interpreted as exploratory and hypothesis-generating. The findings highlight the need for further research to refine treatment strategies, optimize patient selection, and explore the long-term implications of this therapy. A well-designed randomized controlled trial would be instrumental in validating these findings and establishing causal relationships. Addressing these gaps will enable future studies to build on current evidence to improve the management of acute low back pain and enhance patient outcomes.
